# Inhibitory Effect of Dihydroartemisinin on the Proliferation and Migration of Melanoma Cells and Experimental Lung Metastasis From Melanoma in Mice

**DOI:** 10.3389/fphar.2021.727275

**Published:** 2021-09-02

**Authors:** Qi Zhang, Linbo Jin, Quanxin Jin, Qiang Wei, Mingyuan Sun, Qi Yue, Huan Liu, Fangfang Li, Honghua Li, Xiangshan Ren, Guihua Jin

**Affiliations:** ^1^ Department of Immunology and Pathogenic Biology, Yanbian University Medical College, Yanji, China; ^2^ Department of Pathology and Physiology, Yanbian University Medical College, Yanji, China

**Keywords:** dihydroartemisinin, melanoma, metastasis, EMT, tumor microenvironment

## Abstract

Melanoma is aggressive and can metastasize in the early stage of tumor. It has been proved that dihydroartemisinin (DHA) positively affects the treatment of tumors and has no apparent toxic and side effects. Our previous research has shown that DHA can suppress the formation of melanoma. However, it remains poorly established how DHA impacts the invasion and metastasis of melanoma. In this study, B16F10 and A375 cell lines and metastatic tumor models will be used to investigate the effects of DHA. The present results demonstrated that DHA inhibited the proliferative capacity in A375 and B16F10 cells. As expected, the migration capacity of A375 and B16F10 cells was also reduced after DHA administration. DHA alleviated the severity and histopathological changes of melanoma in mice. DHA induced expansion of CD8^+^CTL in the tumor microenvironment. By contrast, DHA inhibited Treg cells infiltration into the tumor microenvironment. DHA enhanced apoptosis of melanoma by regulating FasL expression and Granzyme B secretion in CD8^+^CTLs. Moreover, DHA impacts STAT3-induced EMT and MMP_S_ in tumor tissue. Furthermore, Metabolomics analysis indicated that PGD2 and EPA significantly increased after DHA administration. In conclusion, DHA inhibited the proliferation, migration and metastasis of melanoma *in vitro* and *in vivo*. These results have important implications for the potential use of DHA in the treatment of melanoma in humans.

## Introduction

Melanoma is a malignant tumor with a progressively higher prevalence and worse prognosis worldwide. Melanoma is an aggressive form of skin cancer that often results in early metastasis due to the loss of cell adhesion of the primary tumor, leading to high mortality rates (Ferlay et al., 2015). Furthermore, melanoma is more prone to spread than any other form of skin carcinomas, with the lungs being the most common site of distant metastasis, few satisfactory treatments are available, except for surgery. The FDA presently approved immune checkpoint inhibitors for use in patients with advanced melanoma, such as Nivolumab and Pembrolizumab (Topalian et al., 2019), and immune checkpoint therapy markedly reduced cancer burden and improved both progression-free survival and response rates in patients with advanced melanoma. Nevertheless, patients who ceased CTLA-4/PD-1 blockade therapy due to severe irAEs experienced a higher relapse rate or significant toxicity after resuming anti-PD1 treatment at a relatively high speed (Pollack et al., 2018). Furthermore, the benefits of Anti-PD1 therapy are only temporary, most patients who undergo monotherapy develop drug resistance. While the FDA has also approved several immunotherapies for patients with advanced melanoma, the response rates are generally low, and the severe side effects of immunotherapy can be fatal for patients. Therefore, it is urgent to develop a safer, more effective drug with fewer side effects to gradually inhibit the distant metastatic ability of melanoma and reduce the mortality rate of patients.

Dihydroartemisinin (DHA), the principal artemisinin extracted from the traditional Chinese medicine Artemisia annua, has better water solubility and antimalarial activity compared to other artemisinin derivatives (Lin et al., 1989). In addition, several researchers have demonstrated that DHA is minimally toxic to normal cells (Efferth and Kaina, 2010). It has not only been applied as an antimalarial drug. Still, it has also proven anti-tumor properties, such as lung cancer (Yan et al., 2018), breast cancer (Yao et al., 2018), prostate cancer (Paccez et al., 2019), ovarian cancer (Li et al., 2017). DHA has previously been shown to exhibit toxic effects on various cancer cells by facilitating cell cycle arrest (Lin et al., 1989), promoting apoptosis (Thongchot et al., 2018), preventing angiogenesis (Ho et al., 2014), and abrogating cancer invasion and metastasis (Cheong et al., 2020). Our previous studies demonstrated that DHA inhibits melanoma *in situ* and promotes autophagy (Yu et al., 2020). However, how DHA affects the development of metastatic melanoma is still being explored.

It is widely known that metastasis is a significant factor in tumor mortality, accounting for 90% deaths. Alarmingly, recent studies have indicated that metastases may occur early in melanoma development or before the primary tumor has formed. Currently, only the treatment methods used to inhibit melanoma migration and metastasis are not perfect. Therefore, inhibition of metastasis is considered to be the primary treatment for melanoma.

In the current research, we have closely examined the immunomodulatory activity and anti-tumor capacity of DHA on melanoma using *in vitro* and *in vivo* assays. In addition to demonstrating growth-inhibitory and pro-apoptotic properties of DHA in melanoma cells, our studies show that DHA can stimulate the immune response to melanoma *in vivo*. The activity of DHA on the lymphocyte component of the tumor microenvironment may be a key finding in the tumor immune escape response (Simiczyjew et al., 2020). Therefore, our conclusions disclosed another facet of the inhibitory effects of DHA on melanoma, which might have potential clinical implications.

## Materials and Methods

### The Cell Lines and Cell Culture

The B16F10 cells were purchased from the Fudan IBS Cell Center (China). The A375 cells were a gift from Associate professor Yingshi Piao from the Department of pathology and physiology, Yanbian University, China. All cells were cultured in Dulbecco’s Modified Eagle Medium (DMEM, Gibco, Gaithersburg, MD), supplemented with 10% fetal bovine serum (Gibco, Gaithersburg, MD) 1% penicillin/streptomycin mixture in a humidified 5% CO_2_ atmosphere at 37°C.

### Construction of the Mouse Melanoma Model

8-to-10-week-old female C57BL/6 mice were obtained from the Experimental Animal Centre of Yanbian University. Mice were housed in Specific pathogen Free conditions. The Experimental Animal Ethical Committee approved all studies and procedures of Yanbian University before the initiation of the experiment (SYXK (ji) 2020-0009). The mice were injected via the tail vein with 2 × 10^6^ B16F10 cells in 400 ml of phosphate-buffered saline to build the experimental melanoma lung metastasis model. Dihydroartemisinin was administered to mice by gavage at both high and low doses (25/50 mg/kg/d) every day, and the mice were sacrificed after 28 days of continuous gavage treatment.

### Cell Proliferation Assay

The influence of DHA on the proliferation of B16F10 cells was measured by Cell Counting Kit-8 assay (Dijindo, Japan). B16F10 cells in the mid-log phase were plated at a density of 5 × 10^3^ cells per well for 24 h in a 200 μL medium. The medium was then substituted with DMEM containing different concentrations of DHA (total volume of 200 µL per well). A375 cells were plated in 96-well plates at a density of 1 × 10^5^ cells per well and treated with different concentrations of DHA for 48 h. MTT (5 mg/ml) was added and then incubated at 37°C in the dark for 4 h. The addition of DMSO stopped the reaction. After a further 48 h of incubation, the proliferation rate of the cells was calculated by measuring the absorbance (OD = 450 nm).

### Wound Healing Assay

Cells were seeded on 6 well plates. After the cells have reached 100% confluence, a wound is scraped out on the monolayer using a conventional pipette tip, and the dislodged cells or any loose cell debris is removed by washing with PBS. Different concentration gradients of DHA were added for culture and photographed at 0, 12, 24, and 48 h, respectively.

### Cell Migration Assays

Cell migration assays were performed with transwell inserts (Corning, NY, United States). A375 or B16F10 cells were placed into the upper chamber, and serum below DMEM-1% FBS medium was added as a chemoattractant in the lower chamber. 4–6 h later, different concentrations of DHA were inserted. The cells were incubated for 24 h and then fixated with methanol and stained with saponin solution for 8 min. The migratory situation was known by observing the cells that migrate to the lower side of the filter in a microscope. The experiment was repeated in triplicate.

### H&E Staining and Immunohistochemistry

After 4 weeks, the mice were euthanized, and lung samples were removed for paraffin-embedded cross-sections. These sections were carefully placed in 50°C incubators overnight, and the sections were first stained with hematoxylin and then with eosin. These slides were washed in distilled water and then dehydrated through graded alcohol, dried at room temperature, and observed histopathological changes under the microscope. After dissolving off the wax from the sections with xylene, they were hydrated with graded alcohol. The sections were first rinsed with PBS and exposed to citric acid buffer for 20 min for antigen retrieval. The Ki-67 (1:400), Foxp3 (1:100), CD4 (1:100), and CD8 (1:400) mAb were obtained from Cell Signaling Technology and incubated on sections overnight according to those antibody instructions. The sections were exposed to the appropriate horseradish peroxidase-conjugated secondary antibody and developed in a dark room at the ambient temperature of 10 min. The sections were rinsed with tap water, and the substrate reaction was terminated. The cells were counted from the infection site in five serials sections, the numbers of infiltration cells were averaged in more than five power microscopic fields (HPFs, 0.07 mm^2^).

### Western Blotting Assay

RIPA containing PMSF, protease inhibitor, and phosphotransferase inhibitors were used for protein extraction. Measurement of protein concentrations in lung tissue and cells using a bicinchoninic acid protein assay kit (Solarbio, Beijing, China). Equal amounts of protein were then loaded onto SDS-PAGE mini-gels, run, and transferred onto polyvinylidene difluoride membranes (Roche, Switzerland). These were then blocked with 5% skimmed milk and incubated overnight with the relevant primary antibody. N-cadherin (1:1,000), ZEB1 (1:1,000), Snail (1:1,000), and Twist (1:1,000) mAb were obtained from Proteintech (Rosemont, IL, United States); caspase-3 (1:500), Cleaved-caspase-3 (1:500), caspase-8 (1:1,000), Cleaved-caspase-8 (1:500), *p*-Ezrin (1:1,000), Ezrin (1:1,000), p-STAT3 (1:500), STAT3 (1:500), p-STAT1 (1:500), STAT1 (1:500), *p*-AKT (1:1,000), AKT (1:1,000), p-P65 (1:500) and P65 (1:1,000) mAb supplied by Cell Signaling Technology (Boston, MA, United States); β-actin, Vimentin (1:1,000), E-cadherin (1:1,000), MMP-2 (1:1,000), MMP-9 (1:1,000), *p*-Ezrin (1:1,000) and Ezrin (1:1,000) mAb were provided by Abcam (Cambridge, United Kingdom). Horseradish peroxidase-conjugated secondary antibodies are used for the detection of primary antibodies. Finally, the membranes were scanned using a Bio-Rad Gel imaging system (Bio-Rad, United States) after visualization treatment using the ECL reagent. Protein expressions were analyzed with ImageJ software.

### Enzyme-Linked Immunosorbent Assay

ELISA kits (Mbbiology biological, China) were used to assess the expression levels of AST, ALT, Cr, and β2-MG on the sera collected from each group. Experimental samples were taken in triplicate and repeated three times.

### Immunofluorescence Analysis

Paraffin-embedded cross-sections of lung tissue from each experimental group were processed as described previously. The FITC-conjugated CD8, Cy3-conjugated Fas (1:200, Bioss, Beijing, China) and Cy3-conjugated Granzyme B mAb (1:200, Absin, Shanghai, China) were respectively used and DAPI to visualize the nuclei. Capture images of the cell signals with a fluorescent microscope.

### Statistical Analysis

All data were analyzed with GraphPad Prism 7.0 (GraphPad Software, La Jolla, United States) and exhibited as the means ± SD. The significance of differences was determined using the Student’s *t*-test and one-way ANOVA analysis of variance followed by Dunnett’s test. *p* < 0.05 was considered statistically significant.

## Results

### DHA Inhibits the Proliferation and Migration Ability of Melanoma Cells

**FIGURE 1:**
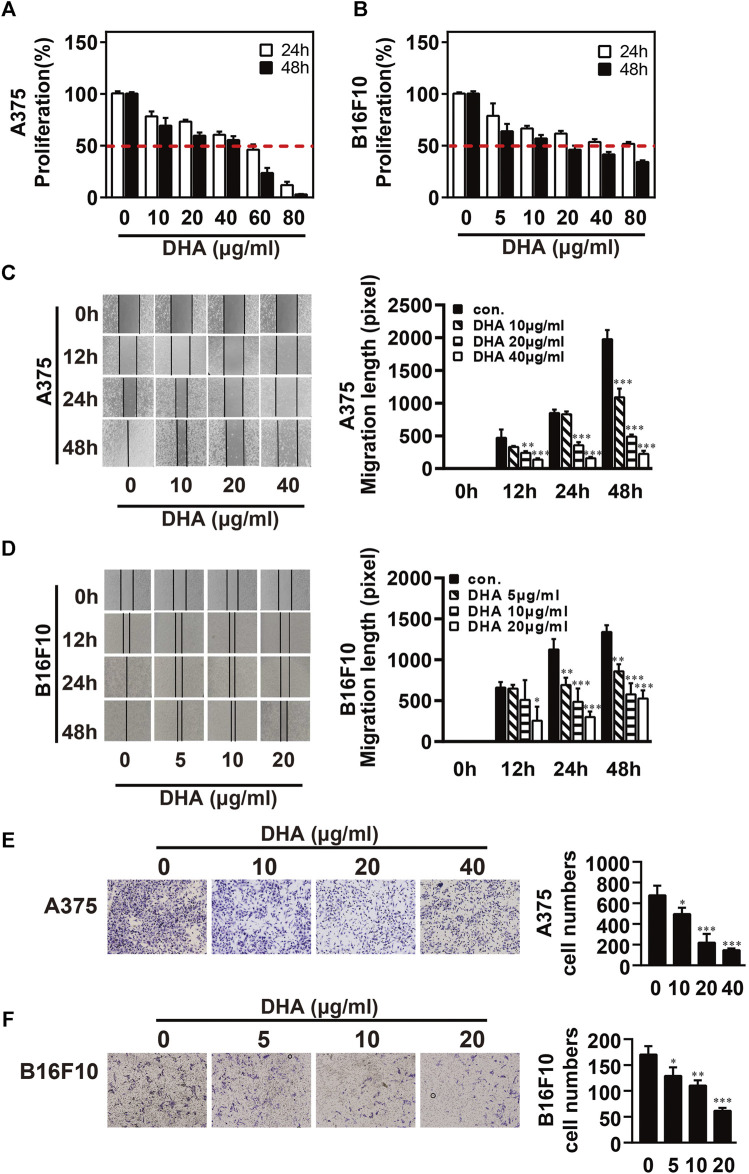
DHA inhibits the proliferation and migration ability of melanoma cells. (**A,B**) Melanoma cells were treated with different concentrations of DHA (A375: 0, 10, 20, 40 μg/ml, B16F10: 0, 5, 10, 20 μg/ml), then cell viability was assessed by MTT assay or CCK-8 assay. (**C,D**) Wound healing assay for demonstrating the inhibitory effect of DHA on the migration of melanoma cells. (**E,F**) Melanoma cells were treated with indicated concentrations of DHA for 24 h, and the invasive capacity was determined by the transwell migration assay. The experiments were repeated at least three times independently, and the data are presented as the mean ± standard deviation (SD) (*p < 0.05; **p < 0.01; ***p < 0.001 compared to control group).

To investigate the inhibitory effect of DHA on melanoma, A375 and B16F10 cells were treated with DHA by different concentrations. Cell proliferative capacity was detected by MTT or CCK-8 assay at 24 and 48 h (Figures 1A,B). The results indicated the viability of A375 and B16F10 cells was markedly reduced in a time- and dose-dependent manner. IC50 values of DHA inhibition in melanoma cells were used in subsequent experiments. To further evaluate the effect of DHA on the migration of melanoma cells, A375 and B16F10 cells were treated with different concentrations of DHA. Wound healing assay indicated that the lateral migration capacity of A375 was markedly weakened in a dose-dependent manner after DHA treatment compared to the control group (Figure 1C). As expected, the migration of B16F10 was also inhibited by DHA-treated groups (Figure 1D). Moreover, similar results were shown by transwell migration assay in A375 and B16F10 cells (Figures 1E,F). These results demonstrated that DHA markedly suppressed the proliferation and migration ability of A375 and B16F10 cells *in vitro*.

### DHA Inhibits Lung Metastasis of Melanoma *in vivo*

**FIGURE 2:**
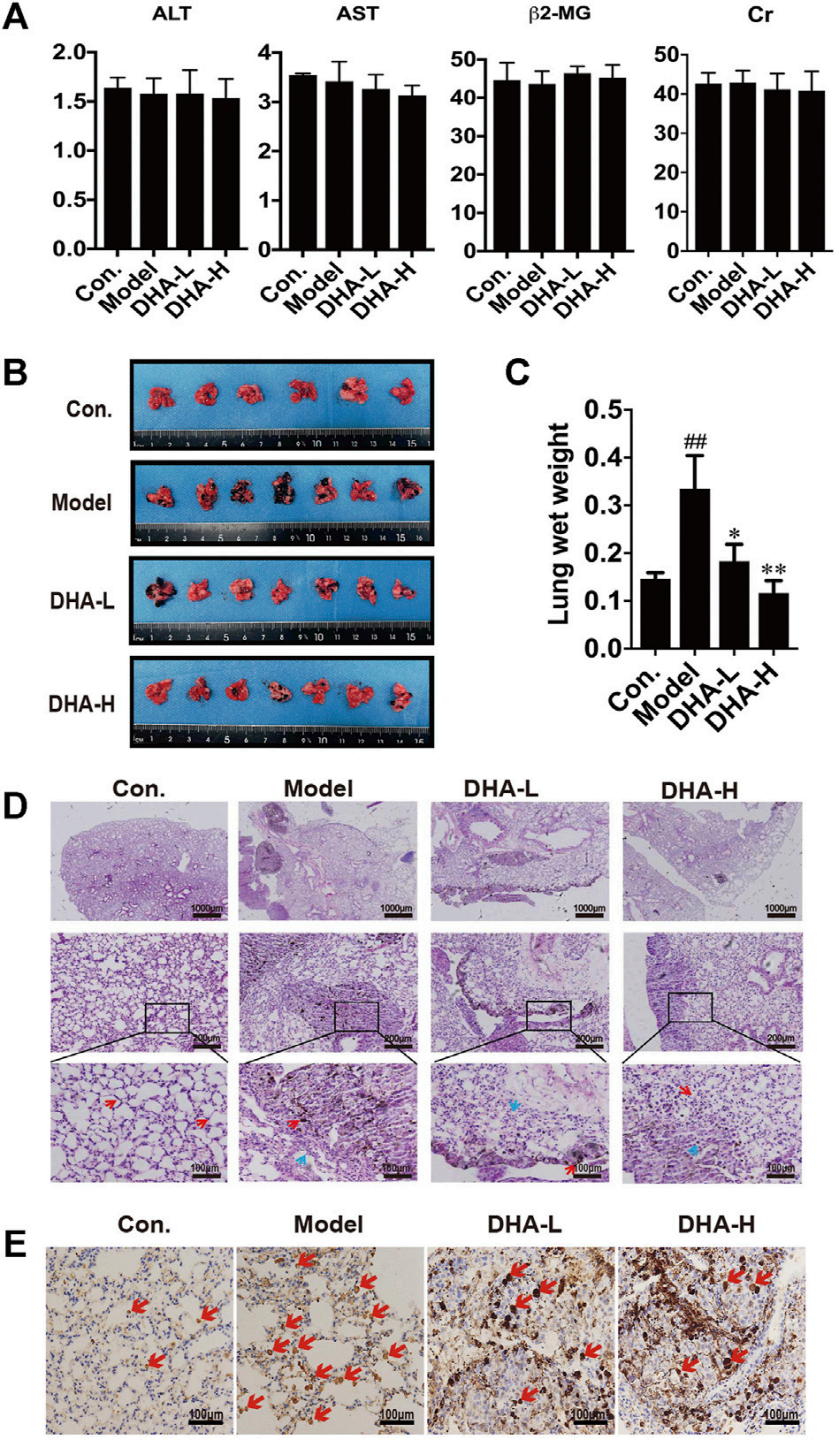
DHA inhibits pulmonary metastasis of melanoma in mice **(A)** Detection of ALT, AST, β2-MG, and Cr of liver function and renal function in mouse serum by ELISA. **(B)** Gross images of metastatic tumor tissues and normal tissues (n ≥ 6). **(C)** The wet lung weight of mice was weighed (^##^ *p* < 0.01 vs control group; ^*^ *p* < 0.05; ^**^ *p* < 0.01 vs model group). **(D)** Immunopathological damage was assessed by hematoxylin and eosin (H&E) staining of the lung tissues. The red arrow indicated melanoma cells of lung metastatic; The blue arrow indicated alveolar duct structure (up×40, scale bars = 1,000 µm; middle×200, scale bars = 200 µm; down×400, scale bars = 100 µm). **(E)** Tumor tissues of lung metastasis were stained with Ki-67 by immunohistochemistry.

To investigate the therapeutic effect of DHA *in vivo*, melanoma lung metastasis mouse models were established. Mice were injected with B16F10 cells via tail vein, and then DHA was administered by daily oral gavage at a dose of 25 mg/kg/day or 50 mg/kg from day 0 to day 28. All mice were observed daily for signs and weighed. The mice were sacrificed 24 h after the last gavage treatment. To examine whether DHA is poisonous to mice, ALT, AST, β2-MG and Cr expression was measured by ELISA. The results showed no significant differences in ALT, AST, β2-MG and Cr before and after DHA administration (Figure 2A). Furthermore, the mouse lungs from the model group were bearing a high burden of metastatic melanoma nodules compared with the control group. Following DHA treatment, the number of melanoma nodules was markedly decreased. It can also be seen that no large metastatic lung nodule was observed in lung tissue after high-dose DHA treatment (Figure 2B). Consistent with the above results, DHA treatment reduced the lung’s wet weight and normalized status (Figure 2C, *p* < 0.05).

The tendency observed for lung metastases was also confirmed by histopathology. In model group, we observed multiple melanoma metastases with many infiltrating melanoma cells. Metastatic melanoma nodules were present throughout the lung, and there were numerous aggregated neoplastic foci. After DHA administration, lung metastasis of melanoma was significantly inhibited. Compared with model group, metastatic melanoma nodules were markedly reduced, accompanied by the disappearance of aggregated melanoma foci and fewer melanoma cells infiltration (Figure 2D). Moreover, the proliferation of melanoma cells was verified by IHC staining. The result showed that DHA treatment significantly reduced Ki67 positive cells in lung tissue (Figure 2E). Collectively, the above results indicated that DHA could alleviate lung metastasis of melanoma in mice.

### DHA Affects T Cell Subsets in Tumor Microenvironment

**FIGURE 3:**
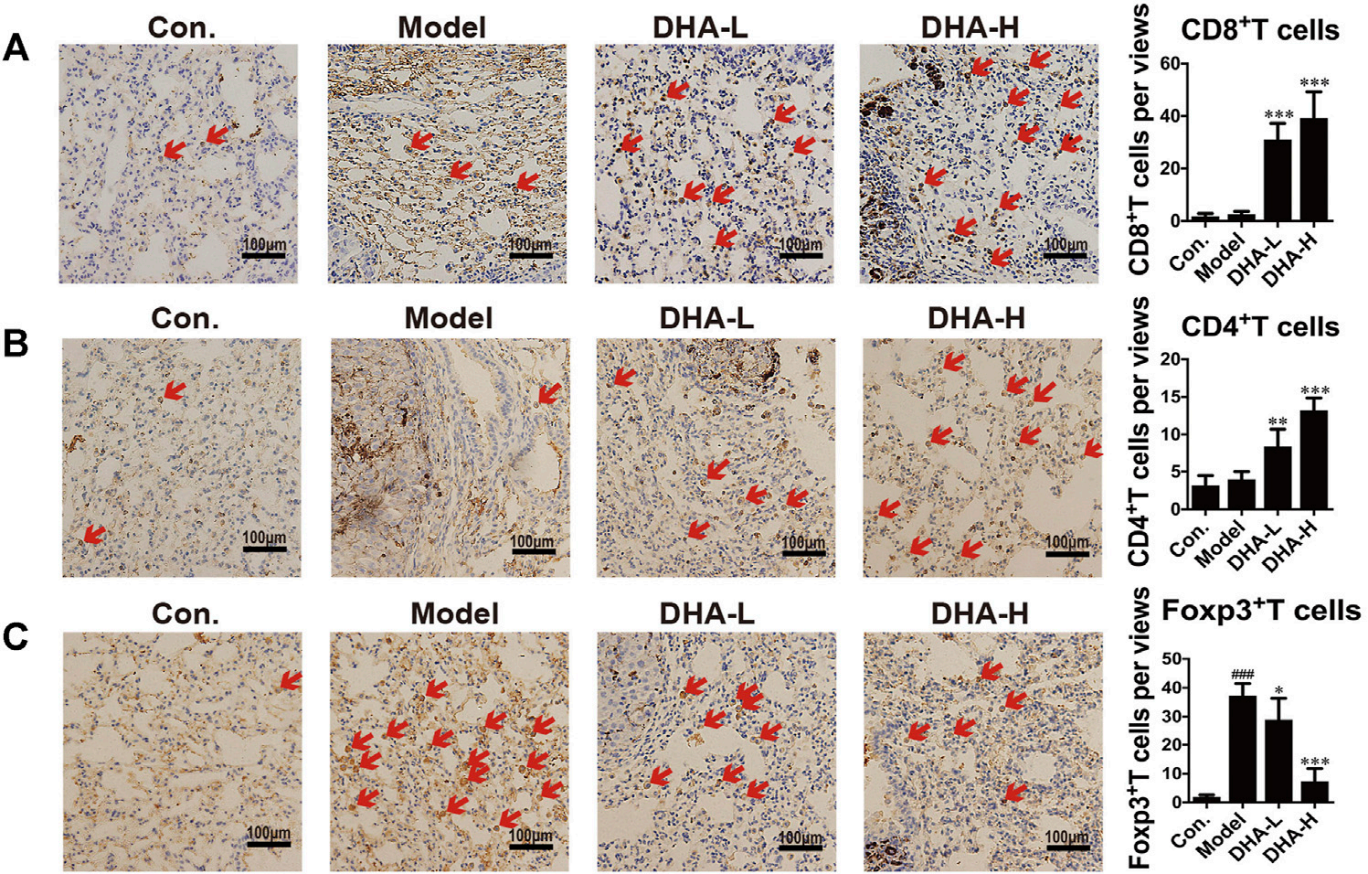
DHA effectively suppresses melanoma severity through enhancing T cell-mediated anti-tumor immune reaction (**A–C**). Tumor tissues of lung metastasis were stained with A: CD8, B: CD4, and C: Foxp3 by immunohistochemical (Original magnification: ×400, scale bars = 100 µm) (^###^ *p* < 0.001 vs control group; ^*^ *p* < 0.05, **p < 0.01, ***p < 0.001 vs model group).

To verify the effect of DHA on T cell subsets in melanoma lung metastasis, IHC staining analysis was performed. Few CD8^+^T cells were present in the model group. On the contrary, normalization in the number of CD8^+^T cells was observed in DHA-treated mice (red arrows), and CD8^+^T cells were distributed in clusters and infiltrated into the tumor microenvironment (Figure 3A). The numbers of CD4^+^T cells were also markedly increased in the tumor microenvironment after DHA treatment (Figure 3B). Moreover, DHA treatment significantly reduced Foxp3^+^cells infiltration into tumor tissues and returned to levels similar to those in the wide-type mice (Figure 3C). Thus, DHA promoted the CD8^+^CTL and suppressed the Treg infiltration into the tumor microenvironment in melanoma mice.

### DHA Enhances the CD8^+^CTLs-Mediated Apoptotic Pathway

**FIGURE 4:**
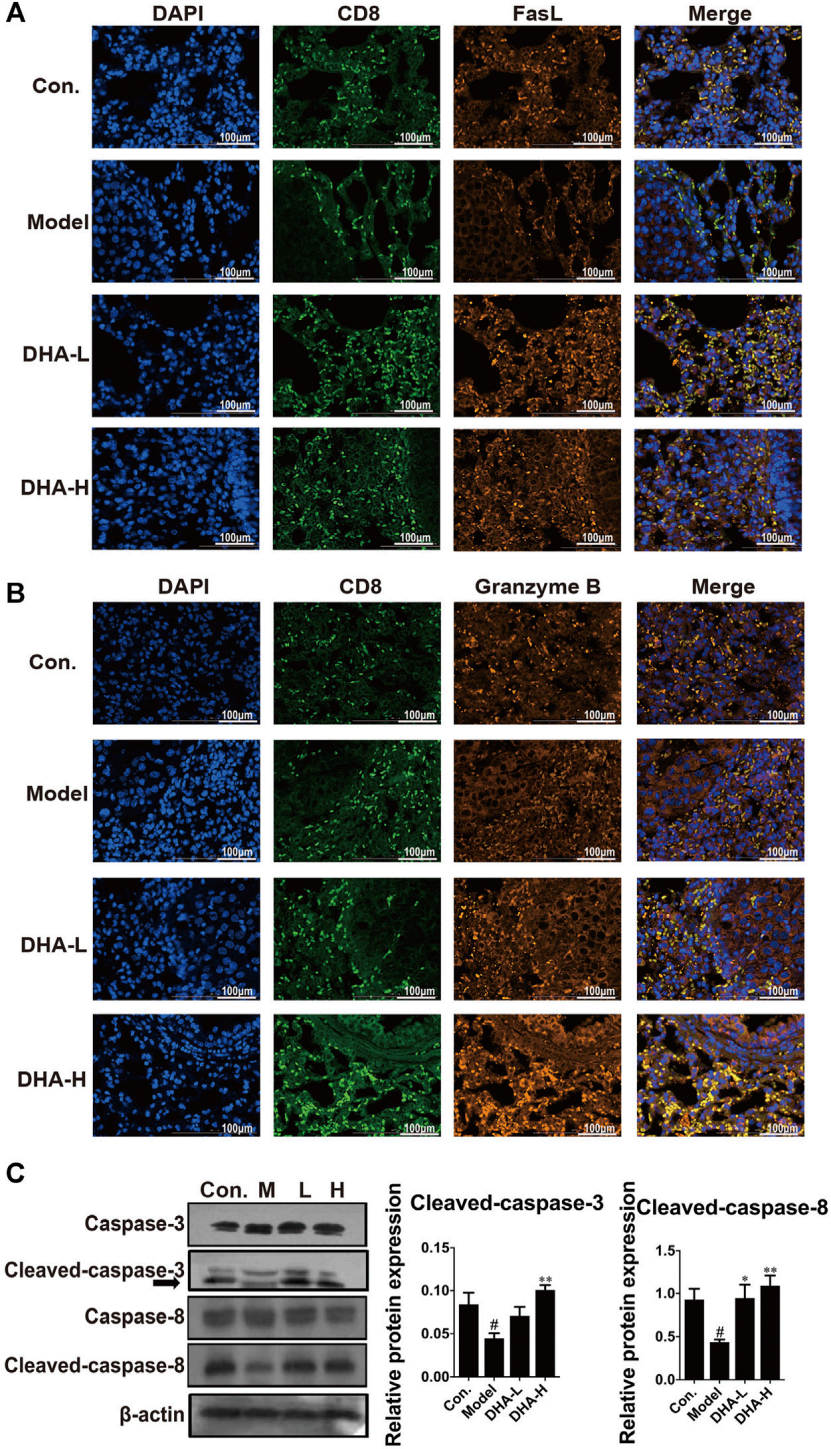
DHA enhances FasL and Granzyme B and activates apoptotic pathways in CD8^+^CTLs. The expression levels of **(A)** FasL^+^CD8^+^T, and **(B)** Granzyme B^+^CD8^+^T before and after DHA treatment (Original magnification: ×400, scale bars = 100 µm). **(C)** Effect of DHA on caspase-related proteins in tumor and normal tissues. The protein expression levels of Cleaved-caspase-3, Caspase-3, Cleaved-caspase-8 and Caspase-8, as determined by Western blotting assay. β-actin was used as the loading control (^#^ *p* < 0.05 vs control group; ^*^ *p* < 0.05, **p < 0.01 vs model group).

Next, we examined the expression of FasL and the secretion of Granzyme B in CD8^+^CTLs by immunofluorescence in tumor tissue. Compared with the control group, there were fewer FasL^+^CD8^+^CTLs cells and Granzyme B^+^CD8^+^CTLs cells in the model group. In contrast, FasL^+^CD8^+^CTLs cells were remarkably up-regulated after DHA treatment (Figure 4A). Moreover, DHA treatment markedly increased the expression of Granzyme B in the CD8^+^CTLs cells (Figure 4B).

In general, FasL-induced apoptosis involves activating caspases responsible for killing tumor cells (Green and Llambi, 2015). To in-depth evaluate the cascade of apoptosis following DHA treatment, the expression of caspase proteins was determined. As expected, compared with the model group, the expression of Cleaved-caspase-3 and Cleaved-caspase-8 were enhanced in a dose-dependent way after DHA treatment (Figure 4C). These results suggested that DHA facilitated apoptosis by modulating the anti-tumor immune function of CD8^+^CTLs.

### DHA Affects Cell Metastasis Through the EMT Process in Melanoma

**FIGURE 5:**
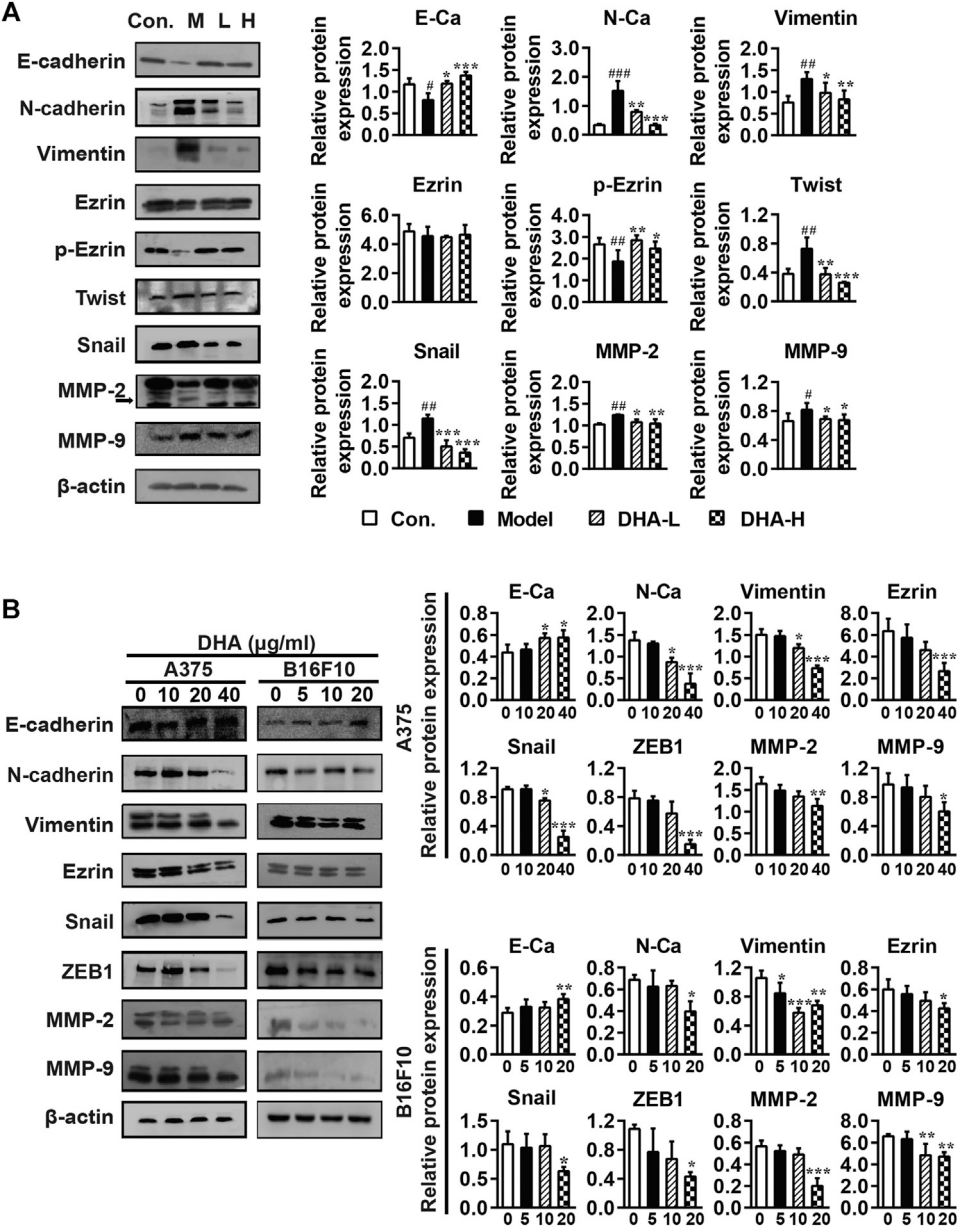
DHA affects cell metastasis through the EMT process in melanoma **(A)** Effect of DHA on EMT and MMPs expression in tumor and normal tissues (^#^ *p* < 0.05, ^##^ *p* < 0.01, ^###^ *p* < 0.001 vs control group; ^*^ *p* < 0.05, **p < 0.01, ***p < 0.001 vs model group). **(B)** Effect of different concentrations of DHA on the expression of EMT and MMPs expression in A375 cells and B16F10 cells (^*^ *p* < 0.05; **p < 0.01; ***p < 0.001 compared to control group). β-actin was used as the loading control.

Tumor cells undergoing EMT phenotypic changes usually involve losing epithelial properties and acquiring mesenchymal properties, reflecting enhanced metastatic and invasive properties (Caramel et al., 2013). To confirm whether the anti-metastatic properties of DHA are mediated by modulating the EMT process, specific proteins of EMT were determined by Western blotting. As shown in Figure 5A, DHA reversed EMT changes in tumor tissues, causing E-cadherin and *p*-Ezrin reinventing and the re-inhibition of Vimentin, N-cadherin, Snail, and Twist expression compared to the model group. Subsequently, tumor metastasis-related proteins MMP-2 and MMP-9 were determined. It was found that the expression levels of MMP-2 and MMP-9 were lower after DHA administration than in the model group.

Besides, the effects of DHA on the expression of EMT and MMPs-related proteins were further demonstrated in A375 and B16F10 cells. Compare with the control group, the expression of E-cadherin was significantly increased, while the expression of Vimentin, Ezrin, N-cadherin, Snail, ZEB1, MMP-2 and MMP-9 were significantly decreased after DHA administration in A375 and B16F10 cells (Figure 5B). We also examined the morphological changes of melanoma cells after administration. It can be revealed that A375 cells are spindle-shaped and polygonal. The A375 cells with DHA treatment attained an epithelial morphology, lost cell polarity, and acquired dispersed morphology. Similarly, it could be observed that B16F10 cells formed pseudopod-like protrusions with robust migration and metastasis ability in the control group, while the DHA-treatment group tended to have the opposite morphology (Supplementary Figure 1). The above results suggested that DHA affects cell metastasis through the EMT process of melanoma *in vivo* and *in vitro.*

### DHA Inhibits Pulmonary Metastases of Melanoma via Affecting STAT1/STAT3 Signaling Pathway

**FIGURE 6:**
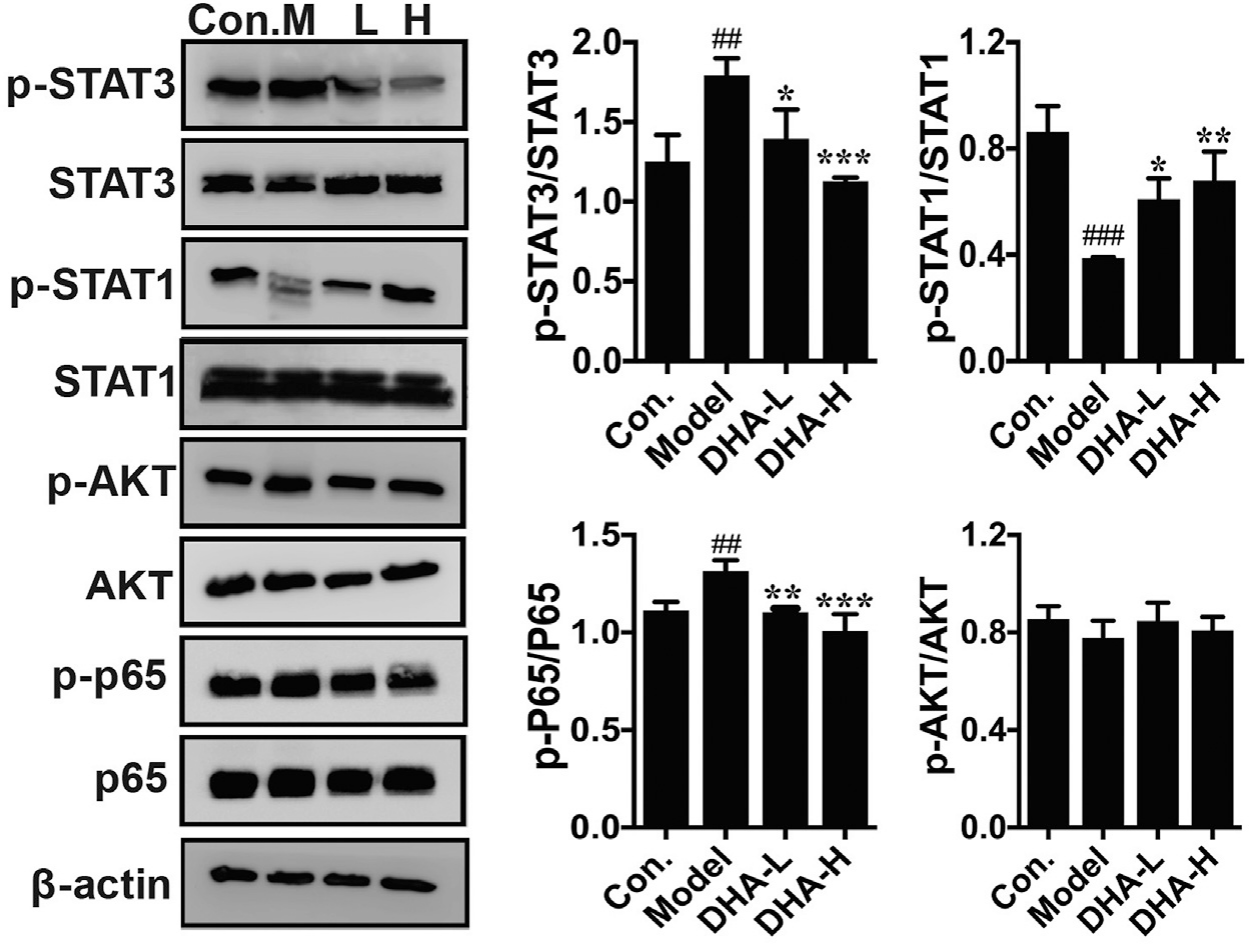
DHA affects the STAT signaling pathway in pulmonary metastases from melanoma. The protein levels of p-STAT3/STAT3, p-STAT1/STAT1, p-Akt/Akt, and p-P65/P65 were detected by western blot assay (^##^ *p* < 0.01, ^###^ *p* < 0.001 vs control group; ^*^ *p* < 0.05, **p < 0.01, ***p < 0.001 vs model group).

The acquisition of an aggressive and motile phenotype by metastatic tumor cells is a prerequisite for the metastatic cascade. At the same time, frequent activation of STAT is associated with EMT initiation, thereby enhancing tumor development and progression. The critical proteins of its associated signaling pathway were examined to clarify the specific signaling pathway through which DHA inhibited melanoma metastasis. The phosphorylation of STAT3 and p65 expression were markedly reduced in the DHA-treated group compared to the model group. By contrast, phosphorylation of STAT1 expression was increased significantly after DHA treatment. However, the expression of *p*-Akt was not remarkably different in the groups (Figure 6). The above results indicated that DHA could infect melanoma lung metastasis by impacting the STAT1/STAT3 signaling pathway.

### DHA Regulates Metabolic Pathways in Lung Metastasis of Melanoma in Mice

**FIGURE 7:**
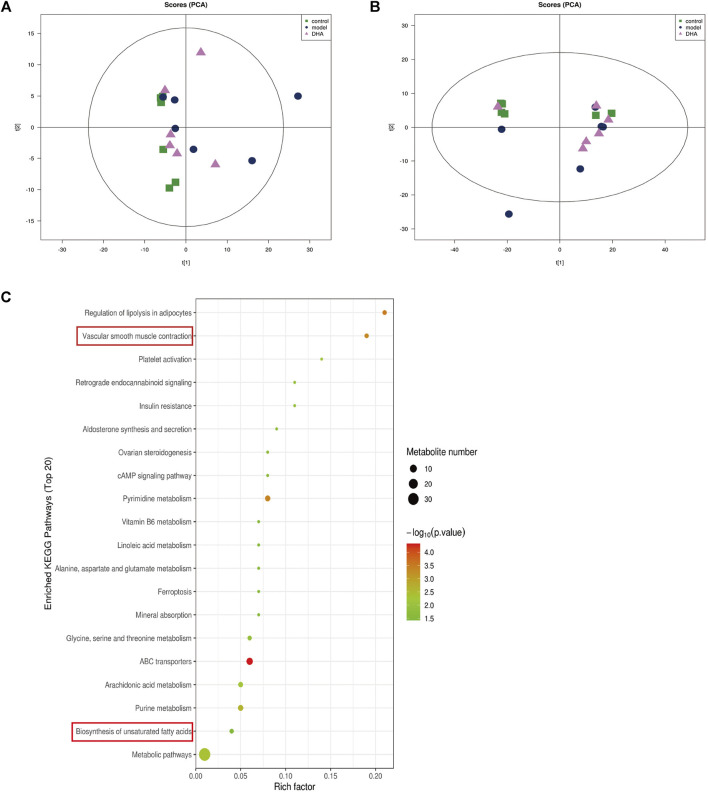
DHA affects cancer tissue metabolism in lung metastasis of melanoma in mice. Mice were sacrificed after 28 days of continuous gavage treatment, and lung tissue was collected for non-targeted metabolomics analysis **(A)** PCA atlas of cancer tissue samples in positive ion mode in each group. **(B)** OPLS-DA atlas of tumor tissue samples in positive ion mode in each group. **(C)** DHA affected metabolic pathways of tumor tissue in mice with melanoma lung metastasis.

Recent studies have demonstrated that metabolism is significantly associated with tumorigenesis and metastasis (Luengo et al., 2017; Kosmopoulou et al., 2020; Li et al., 2016). Based on the strategy of tumor immunotherapy with metabolic regulation, UHPLC-Q-TOF-MS technology was used. The PCA method used the corrected positive and negative ion data to explore lung tissue metabolic spectrum changes. The results showed that the groups were quite different, and each group was separated (Figures 7A,B). Through untargeted metabolomic analysis, 43 metabolites differentiators were found (Table 1). Among them, compared with the control group, 5 notable metabolites (1-Palmitoylglycerol, Adenine,.gamma. -L-Glu-. epsilon. -L-Lys, UDP-N-acetylglucosamine, D-Erythrose 4-phosphate) were significantly increased in the model group, while DHA treatment markedly downgraded the 1-Palmitoylglycerol, Adenine,.gamma. -L-Glu-. epsilon. -L-Lys, UDP-N-acetylglucosamine, and D-Erythrose 4-phosphate expression levels. By contrast, 7 notable metabolites such as Eicosapentaenoic acid (EPA), Arachidonic Acid, (+-)5,6-DHET, D-Pipecolinic acid, Acetylcarnitine, 3′-O-methylguanosine, Prostaglandin D2 (PGD2) were significantly decreased in the model group than that control group, while DHA administration markedly up-regulated the 7 notable metabolites expression levels (data not shown). PGD2 was found to be a potent inhibitor of melanoma cell replication *in vitro* (Stringfellow and Fitzpatrick, 1979; Bhuyan et al., 1986), and EPA has proved a potential anti-melanoma metabolite (Pappalardo et al., 2015). In the current study, the KEGG pathway enrichment map showed that PGD2 belongs to the Vascular smooth muscle contraction metabolic pathway and EPA belongs to the Biosynthesis of unsaturated fatty acids metabolic pathway, the *P*. values of the KEGG profile shown that they are significantly correlated (Figure 7C). The results indicated that non-targeted metabolomics could screen out the differential metabolites in lung tissues after DHA treatment and understand the involved pathways through the differential metabolites, which makes a preliminary screening for our subsequent experiments on specific targets of DHA treatment of melanoma lung metastasis, as well as for the next step in our experimental program.

**TABLE 1:** The non-targeted metabolomics analysis identified 43 differential metabolites, focusing on Prostaglandin D2 and Eicosapentaenoic acid, marked with red arrows.

Description	ANOVA *p* value
2-Methylguanosine	0.001826042
(+-)5,6-DHET	0.003015182
N1-Methyl-2-pyridone-5-carboxamide	0.003657503
D-Pipecolinic acid	0.005241604
4-Pyridoxic acid	0.005896058
3-methylcytidine	0.00643569
Acetylcarnitine	0.006891939
Guanidoacetic acid	0.007484157
Cytosine	0.011148754
3′-0-methylguanosine	0.013021719
1-Palmitoylglycerol	0.013179646
Cytidine 5′-diphosphocholine (CDP-choline)	0.013194002
Pregnenolone	0.013757724
Adenine	0.016661586
His-Glu	0.016953847
.gamma.-L-Glu-.epsilon.-L-Lys	0.020122791
Riboflavin	0.021477963
Prostaglandin D2 (PGD2)	0.02262804
20-Hydroxyarachidonic acid	0.022757126
Ser-Asp	0.022846151
L-Palmitoylcarnitine	0.022903111
5-Hydroxytryptophol (5HTOL)	0.024863181
Inosine 5′-monophosphate (IMP)	0.025194604
Stearidonic Acid	0.027512153
L-Glutamine	0.027659787
Eicosapentaenoic acid	0.029270988
UDP-N-acetylglucosamine	0.030186028
S-Lactoylglutathione	0.03107692
Pantothenate	0.031456369
1-Myristoyl-sn-glycero-3-phosphocholine	0.033852779
Linoleic acid	0.035036719
Adenosine	0.035462864
Cytidine	0.036464663
Betaine	0.038988825
1-Methylhistamine	0.039300498
L-Alanine	0.03973406
16-Hydroxypalmitic acid	0.041819631
1-Palmitoyllysophosphatidylcholine	0.042158527
Xanthine	0.042925125
Arachidonic Acid (peroxide free)	0.044835414
Betaine aldehyde	0.04668166
Prostaglandin l2	0.047062605
D-Erythrose 4-phosphate	0.047640488

## Discussion

At present, more and more studies focus on elucidating the molecular mechanism of DHA’s anticancer activity. The advantages of DHA as a new class of anti-tumor agents are evident, including good tolerance, low cross-resistance (Sá et al., 2018), low toxicity to normal tissues and cells (Xu et al., 2016), and synergistic with many other conventional anti-tumor drugs (Feng et al., 2014). Oral DHA is rapidly absorbed in the gastrointestinal tract and reaches Cmax, approximately 1–2 h after administration (Dai et al., 2019). The multiple bioactivities of DHA in cancer include inhibition of tumor proliferation, promotion of apoptosis, and suppression of angiogenesis and tumor metastasis. Studies suggested that DHA mediates the microRNA-mRNA regulatory network to promote both apoptosis and angiogenesis in pancreatic cancer (Li et al., 2016). In addition, DHA impedes colony formation, proliferation and induction of ferry death in lung cancer cells by blocking the PRIM2/SLC7A11 axis (Yuan et al., 2020). In our research, DHA suppressed the proliferation, migration and metastasis of melanoma cells *in vitro* and decreased the severity of melanoma and histopathological changes in the lungs of mice. Furthermore, DHA treatment significantly reversed the EMT changes in melanoma cells, affecting the expression of the associated proteins**.**

EMT has a determinant role in tumorigenesis and metastasis (Floor et al., 2011). DHA inhibited EMT of ovarian cancer, thereby reducing its metastasis to the lung, liver and intestine (Bao, 2011; Liu et al., 2018). In breast cancer, DHA inhibits EMT by reducing TGF-β production and decreasing phosphorylation of Smad2 and Smad3 (Li et al., 2020). In a similar vein, Ju *et al.* observed that DHA inhibited breast cancer metastasis by reducing the production of MMPs, TGF-β, and vascular endothelial growth factor (VEGF) (Ju et al., 2018). It has been confirmed that activation of STAT3 could induce EMTs and MMPs and may cause a progressive shift in cell phenotype towards a mesenchymal morphology with implications for the invasive, intravascular and extravascular steps of the metastatic cascade (Rokavec et al., 2014). In this study, treatment with DHA significantly reduced STAT3 phosphorylation. In addition, Wang *et al.* found that IFNα2b markedly regulated the STAT1/STAT3 balance in host lymphocytes and melanoma cells. The pSTAT1/pSTAT3 ratio in tumor cells could be a potent predictor for assessing the clinical treatment of melanoma (Wang et al., 2007). Furthermore, Khan *et al.* demonstrated that overexpression of the ACE C domain in macrophages induced their differentiation into a distinct M1 phenotype in response to melanoma stimulation and increased activation of NF-κB and STAT1, which blocked the activation STAT3 and STAT6 (Khan et al., 2019). Interestingly, in our study, phosphorylation of STAT3 and p65 expression were markedly reduced in the DHA group. By contrast, phosphorylation of STAT1 expression was significantly enhanced after DHA treatment. In addition, DHA also has been shown to inhibit the development of non-small cell lung cancer metastasis by inhibiting the NF-κB/GLUT1 axis (Jiang et al., 2016) and has been demonstrated to enhance the anti-pancreatic cancer potential of gemcitabine via inhibition of the NF-κB axis (Wang et al., 2010). Therefore, we suggest that DHA suppressed melanoma invasiveness by reducing MMPs and EMT activity and expression through inhibiting the constitutive activities STAT3/NF-κB and accelerating the STAT1.

Activated CTLs are the most potent immune effector cells in the body, exerting a robust anti-tumor immune response through various pathways. CTLs act by producing anti-tumor cytokines like perforin and granzyme or by-products killing tumor cells via the Fas/FasL pathway. In pancreatic cancer, Zhou *et al.* found that DHA enhanced the activity of T cells and promoted the secretion of perforin, Granzyme B, and IFN-γ (Zhou et al., 2013). Fas and its receptors FasL and caspase-3/8 are components of the pathway that controls the acceptance of apoptosis in tumor cells (Nagata and Golstein, 1995). Apoptosis can be actuated by extrinsic pathways, which include the enactment of cell surface death receptors, or intrinsic pathways, in which modifications within the astuteness of the mitochondrial membrane initiate the discharge of cytochromes. These pathways focalize at the level of effector caspases. When activated, the effector caspases then cleave the cytoskeleton and nuclear proteins, such as PARP, thus starting the cytolytic program (Fulda and Debatin, 2006). Yeo *et al.* found that DHA promotes caspase-dependent apoptosis of hepatocellular carcinoma SK-Hep-1 cells by a specific protein 1 pathway (Im et al., 2018). Similar to our results, treatment with DHA markedly activated FasL, cleaved caspase-3, and cleaved caspase-8 in the lung metastasis model, supporting the assumption that tumor death occurred in a manner consistent with apoptosis in this study. In addition, the balance of Treg/CTLs was significantly associated with anti-tumor immunity. Treg suppresses the pernicious effect of CTLs by secreting IL-10 and TGF-β, thus realizing tumor immune escape (Ohue and Nishikawa, 2019). It has been shown that DHA inhibits the production of TGF-β (Yao et al., 2018; Li et al., 2020) and TNF-α (Su et al., 2019). Zhang *et al.* revealed that DHA could enhance the proportion of Treg and CD8^+^T cells and decrease the expression of proinflammatory cytokines (Zhang et al., 2020). Noori *et al.* observed that, in pancreatic cancer, DHA had the function of inhibiting Treg and increasing IFN-γ production (Noori and Hassan, 2011). Therefore, it is likely possible that DHA influences Treg/CD8^+^CTL cells’ balance and inhibits the induction of caspase-dependent apoptosis in the tumor microenvironment. The utility of cytokines in melanoma lung metastasis is to be further examined.

The tumor microenvironment is always accompanied by metabolic processes different from normal cells. Batista *et al.* used nuclear magnetic resonance to study serum metabolomics from patients with cirrhosis and hepatocellular carcinoma and found many metabolites involving multiple metabolic pathways (Batista et al., 2018). In our experiment, Metabolomics analysis indicated that PGD2 and EPA significantly increased after DHA administration, which is closely related to suppressing tumors metastasis (Table 1). Shyu *et al.* have revealed that PGD2 was co-localized with H-rev107, suppressed testis cell migration and invasion by the PGD2-cAMP-SOX9 signal pathway (Shyu et al., 2013).

Furthermore, PGD2 levels in cystadenocarcinoma of the ovary have been demonstrated to impede the growth of ovarian cancer cells *in vitro* and *in vivo* and prolong the survival of nude mice with these tumors (Kikuchi et al., 1986). Furthermore, Stringfellow *et al.* found that the PGD2 formed by malignant melanoma cells *in vitro* was inverse proportion to their metastatic potentials. PGD2 also controls pulmonary metastasis of malignant melanoma (Pappalardo et al., 2015). EPA belongs to a family of long-chain n-3 polyunsaturated fatty acids, commonly used as a nutritional supplement to treat tumor-related cachexia (Sharma et al., 2005). It was previously proposed that the EPA can directly influence tumor development, inhibit tumor cell migration, and promote apoptosis, thereby affecting tumor metastasis. Wan *et al.* have shown that EPA induces growth suppression on epithelial ovarian cancer cells through inhibiting PPAR and p53 overexpression (Wan et al., 2016). Yamada *et al.* indicated that the EPA interacted with the activated C kinase 1 receptor and inhibited melanoma cell proliferation via protein kinase C signaling (Yamada et al., 2019). Our dates indicated that PGD2 and EPA were markedly reduced in the model group, but there was a regression trend after DHA administration. These results suggest that DHA treatment for lung metastasis of melanoma may be related to disturbance of metabolism *in vivo* to some extent. Due to the limitations of untargeted metabolomics, this experiment only preliminarily revealed the possible biological material basis of melanoma lung metastasis, suggesting that DHA inhibition of melanoma lung metastasis may be related to amino acid metabolism, fatty acid metabolism, and other small molecule substance metabolism. However, whether the metabolism of other body fluids or tissues, such as blood, urine, and liver, is different or related to lung tissue metabolism in the lung metastasis model of melanoma requires further testing.

In summary, our study shows that DHA has anti-proliferative and anti-metastatic effects on melanoma cells, and confirms these effects in a mouse model of lung metastasis. Our results provide the experimental basis for the application of DHA in the treatment of melanoma and as a valuable drug for the treatment of metastatic melanoma.

## Data Availability

The original contributions presented in the study are included in the article/Supplementary Material, further inquiries can be directed to the corresponding authors.
