# Supplementary figures and images for: Inhibitory Effect of Dihydroartemisinin on the Proliferation and Migration of Melanoma Cells and Experimental Lung Metastasis From Melanoma in Mice
Source: Front Pharmacol. 2021 Sep 2;12:727275. doi: 10.3389/fphar.2021.727275 (PMC8443781; doi:10.3389/fphar.2021.727275)

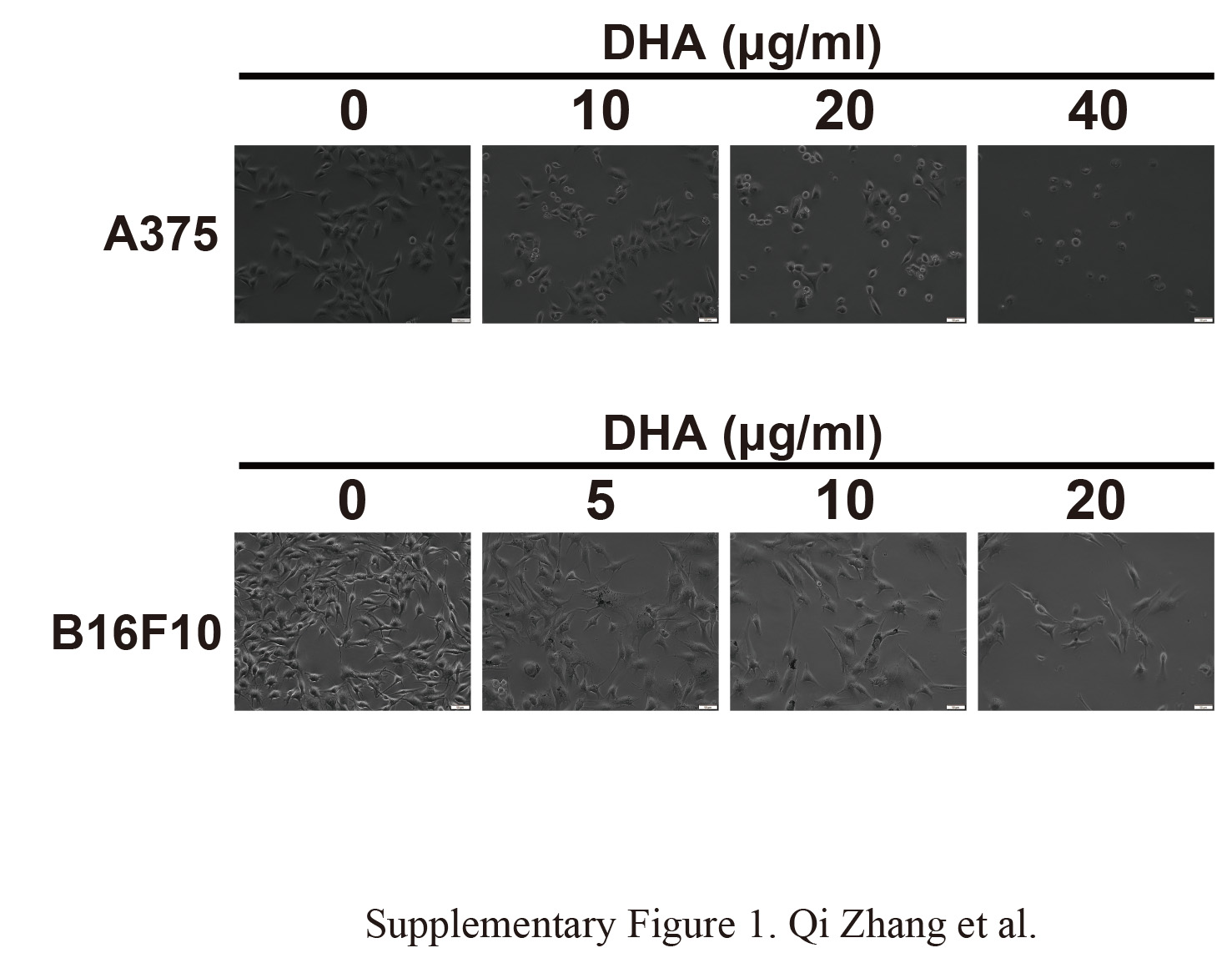

Supplement: Supplementary file 1 [file Image1.JPEG]
